# Tracking Difference in Gene Expression in a Time-Course Experiment Using Gene Set Enrichment Analysis

**DOI:** 10.1371/journal.pone.0107629

**Published:** 2014-09-30

**Authors:** Pui Shan Wong, Michihiro Tanaka, Yoshihiko Sunaga, Masayoshi Tanaka, Takeaki Taniguchi, Tomoko Yoshino, Tsuyoshi Tanaka, Wataru Fujibuchi, Sachiyo Aburatani

**Affiliations:** 1 CBRC, National Institute of AIST, Tokyo, Japan; 2 Center for iPS Research and Application, Kyoto University, Kyoto, Japan; 3 Institute of Engineering, Tokyo University of Agriculture and Technology, Tokyo, Japan; 4 JST, CREST, Sanbancho 5, Chiyoda-ku, Tokyo, Japan; 5 Mitsubishi Research Institute, Inc., Tokyo, Japan; University of North Carolina at Charlotte, United States of America

## Abstract

*Fistulifera* sp. strain JPCC DA0580 is a newly sequenced pennate diatom that is capable of simultaneously growing and accumulating lipids. This is a unique trait, not found in other related microalgae so far. It is able to accumulate between 40 to 60% of its cell weight in lipids, making it a strong candidate for the production of biofuel. To investigate this characteristic, we used RNA-Seq data gathered at four different times while *Fistulifera* sp. strain JPCC DA0580 was grown in oil accumulating and non-oil accumulating conditions. We then adapted gene set enrichment analysis (GSEA) to investigate the relationship between the difference in gene expression of 7,822 genes and metabolic functions in our data. We utilized information in the KEGG pathway database to create the gene sets and changed GSEA to use re-sampling so that data from the different time points could be included in the analysis. Our GSEA method identified photosynthesis, lipid synthesis and amino acid synthesis related pathways as processes that play a significant role in oil production and growth in *Fistulifera* sp. strain JPCC DA0580. In addition to GSEA, we visualized the results by creating a network of compounds and reactions, and plotted the expression data on top of the network. This made existing graph algorithms available to us which we then used to calculate a path that metabolizes glucose into triacylglycerol (TAG) in the smallest number of steps. By visualizing the data this way, we observed a separate up-regulation of genes at different times instead of a concerted response. We also identified two metabolic paths that used less reactions than the one shown in KEGG and showed that the reactions were up-regulated during the experiment. The combination of analysis and visualization methods successfully analyzed time-course data, identified important metabolic pathways and provided new hypotheses for further research.

## Introduction

The search for sustainable and environmentally-friendly fuel is a burgeoning field in biology because organic waste products and organisms are abundant and renewable sources of biofuel compounds. There is strong focus on producing biofuel from food crops, such as corn and soy, as well as oleaginous algae, such as *Chlamydomonas reinhardtii* and *Nannochloropsis oceanica*. One of the big advantages of algae over terrestrial crops is that they require less land to grow on while producing more biomass [1]. This characteristic is important in large-scale production to minimize competition with the production of food or with the preservation of neighboring habitats. Algae can be farmed in open tanks or closed columns and does not deplete soil for agricultural use. Most oleaginous algae accumulate biofuel compounds in low nitrogen conditions at the expense of cell growth [2] [3] [4]. For that reason, we have focused our analysis on a newly sequenced strain of microalgae, *Fistulifera* sp. strain JPCC DA0580, which is able to accumulate lipids while undergoing logarithmic growth [5]. *Fistulifera* sp. strain JPCC DA0580 is a pennate diatom that is possibly an allodiploid, sharing many of its genes with the diatoms, *Phaeodactylum tricornutum* and *Thalassiosira pseudonana*. It demonstrates a high growth rate concurrently with achieving high lipid content (40–60% w/w) [6]. There have been 20,618 genes sequenced from the nuclear, chloroplast and mitochondrion genomes. Although the *Fistulifera* sp. strain JPCC DA0580 genome contains some genes that are homologous to the ones involved in lipid metabolism, the cellular mechanisms for its ability to simultaneously grow and accumulate lipids is unknown.

In our analysis, we utilized RNA-Sequencing (RNA-Seq) data gathered from *Fistulifera* sp. strain JPCC DA0580 while it was grown in oil accumulating and non-accumulating conditions at four time points, from 0 to 60 hours. RNA-Seq is a high-throughput sequencing method that produces a large amount of data per experiment and can be used to investigate differences in gene expression between several conditions. The method produces count data of RNA sequences which can be normalized using Reads Per Kilobase Per Million (RPKM). The normalization corrects for the varying coverage a sequence may get due to its length. Most analyses that involve comparisons in gene expression focus on identifying differentially expressed genes, especially methods that use linear modeling which take advantage of preexisting microarray analyses [7] [8]. Another type of method that is less stringent is gene set enrichment analysis (GSEA), which is more focused on relating the results with previous knowledge. GSEA approaches the data analysis by looking for associations between predefined groups of genes, a gene set, and a phenotype of interest. This type of method is better at detecting small but coordinated differences in gene expression than linear modeling and is less interested in differentially expressed genes and more focused on a group of genes being expressed differently from the background expression. GSEA generally has simple requirements for the data to be analyzed. The important elements are sets of genes that can be compared to the data and data values that can be distilled into one value per gene, usually gene expression or fold change. This makes GSEA more suitable for analysing our data.

There are a variety of GSEA tools available for analyzing high-throughput sequencing data from experiments investigating two conditions with a robust number of replicates on a model organism [9]. For example, online services such as DAVID [10] [11], FuncAssociate [12] and GOEAST [13], statistical packages for R such as SPIA [14] and standalone scripts such as PAGE [15]. Unfortunately, our data was not suitable for these methods. When investigating multiple time points with a new organism, it is sometimes not feasible to have enough replicates, even with the decreasing cost of RNA-Seq experiments. There are some methods that can accommodate these data but they still depend on variance estimation which is inadequate for our data. Therefore, we proposed a new approach to analyse data from a new organism that takes into account the change in gene expression through time in order to avoid reducing our data as done by some existing tools.

We demonstrate a modified approach to GSEA that is able to analyse one sampled data with multiple time points, and custom annotations in an investigation on the difference in gene expression between two conditions through four time points. We then use the results to identify a sequence of reactions starting with a compound such as glucose, and ending with a compound of interest such as triacylglycerol. To create gene sets for a genome with custom annotations, we associate our genes with known KEGG pathways and make each metabolic pathway a gene set. In order to fully utilize the time-course data, each time point is treated as a variable so that GSEA is performed in multiple dimensions, and gene expression variation across time can be conserved. We use re-sampling to address the low replicate number issue and create an empirical cumulative distribution that is then used to calculate the enrichment p-value on multidimensional data without the need to assume multivariate normality. Finally, we visualize and interpret the results using graphs that join the enriched gene sets. The graphs also let us calculate a hypothesized pathway of reactions from one compound to another. In the interest of learning about oil accumulation, we chose to focus our demonstration on the reactions involved in turning glucose into the target biofuel lipid, triacylglycerol (TAG).

## Results and Discussion

### Gene Set Enrichment Analysis

Using the modified GSEA method on our data, we identified 9 significantly enriched pathways (Table 1). These pathways contain genes whose difference in gene expression was significantly different, as a group, to the general background level of gene expression of the whole data set.

**Table 1:** Results of GSEA Method.

Pathway Name	P-value
Photosynthesis	0*
Photosynthesis - antenna proteins	0*
Pentose phosphate pathway	0*
Carbon fixation in photosynthetic organisms	0*
Fatty acid biosynthesis	0*
Amino sugar and nucleotide sugar metabolism	0.013
Methane metabolism 00680	0.013
Oxidative phosphorylation	0.026
Glycolysis	0.026

The enriched pathways identified using GSEA and their enriched p-values. There were 9 pathways enriched out of 39 pathways tested.

*P-value <0.0001.

The photosynthesis and photosynthesis antenna protein pathways were two related pathways that were significantly enriched with p-values <0.0001. The gene expression in the photosynthesis pathway showed a positive relationship between log fold change and time, indicating that there was increased energy synthesis via photosynthesis during oil accumulation. Although a similar relationship was present in the photosynthesis antenna proteins pathway, the log fold change values at 60 hours was higher than in the photosynthesis pathway. Further investigation reveals that the values came from the expression of light-harvesting complex I chlorophyll a/b binding proteins; LHCA1, LHCA2 and LHCA4. Additionally, the general difference in expression of proteins in light-harvesting complex II is lower than in light-harvesting complex I. The preference of light-harvesting complex I may be due to the highly efficient nature of photosystem I [16] even though *Fistulifera* sp. strain JPCC DA0580 is using both systems simultaneously in this case.

The other prominent pathways are related to cellular energy metabolism; glycolysis, the pentose phosphate pathway and oxidative phosphorylation were significantly enriched in our analysis. The glycolysis and pentose phosphate pathways are fundamental to the conversion of glucose to fatty acids while oxidative phosphorylation is essential for providing the energy needed to power metabolic reactions. Some of the proteins in the oxidative phosphorylation pathway form the membrane protein V-type ATPase. It is a proton pump responsible for ATP turnover in mitochondria and was up-regulated in our data. There is some evidence of a relationship between increased C16-C18 length fatty acids, which are used in TAG production, and increased hydrolytic activity of V-ATPase [17]. Along with a gradual down-regulation of NADH dehydrogenase, it would seem that *Fistulifera* sp. strain JPCC DA0580 focuses on recycling ATP instead of reducing NADP+ for its energy requirements during oil accumulation. Predictably, most glycolysis genes were up-regulated during the experiment, although there were notable exceptions; phosphoglucomutase (PGM), phosphoglycerate kinase (PGK) and glyceraldehyde 3-phosphate dehydrogenase (GAPDH). PGM transfers a phosphate group to and from the 1' position to the 6' position in 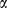-D-glucose so its down-regulation suggests that *Fistulifera* sp. strain JPCC DA0580 is getting its source of 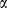-D-glucose 6-phosphate elsewhere. PGK and GAPDH are used in two reversible reactions to make glycerate 3-phosphate which is a key molecule for TAG production [18]. However, this reaction can be done in one irreversible step by glyceraldehyde-3-phosphate dehydrogenase (NADP) which was up-regulated in our data. The substrate for that reaction, glyceraldehyde 3-phosphate, is used in the pentose phosphate shunt to make nucleic and amino acids like deoxyribose, 2-Deoxy-D-ribose 1-phosphate and D-ribulose 5-phosphate. The genes involved in those reactions were found to be up- regulated in our data; they were ribokinase (rbsK), phosphopentomutase (PGM2), 6-phosphogluconate dehydrogenase (PGD) and 3-hexulose-6-phosphate synthase (hxlA). So it seems that *Fistulifera* sp. strain JPCC DA0580 relies on glucose to produce TAG, and nucleic and amino acids to achieve accumulation and growth at the same time while using a proton pump to power the reactions under low nitrogen conditions.

The other significant pathways are related to synthesizing the materials for TAG and growth; they are fatty acid biosynthesis and amino sugar and nucleotide sugar metabolism. Expectedly, the difference in gene expression in fatty acid biosynthesis shows a general up-regulation of the genes in the pathway as *Fistulifera* sp. strain JPCC DA0580 accumulates TAG and continues cell growth. Gene expression in the amino sugar and nucleotide sugar metabolism pathway also had a positive trend through time. The up-regulation of genes in this pathway suggests that sugars are being metabolised for growth during oil accumulation. Two of the up-regulated genes are glucokinase (glk) and glucose-6-phosphate isomerase (GPI) which are involved in reversible reactions that convert glucose into fructose and eventually lead to the production of nucleotide sugars. As the reactions are reversible, we are unable to discern whether the forward or backward reaction was dominant without further data but their up-regulation means that there was a considerable amount of converting occurring.

The next significantly enriched pathway, carbon fixation in photosynthetic organisms, has several genes that are also present in pyruvate metabolism, glycolysis and the pentose phosphate pathway. The genes that exhibit varied differences in gene expression are the ones associated with pyruvate metabolism. During the experiment, malate dehydrogenase (decarboxylating) up-regulated the reaction that turns malate into pyruvate. In contrast malate dehydrogenase (oxaloacetate-decarboxylating) was down-regulated. The preference for the decarboxylating reaction could be due to the reactant, NADP, being used in other reactions, such as photosynthesis. Notably, the pyruvate metabolism pathway was not significantly enriched as a gene set however it only shares seven reactions with the carbon fixation in photosynthetic organisms pathway and is directly linked to 13 other pathways. It is likely that the process of oil accumulation uses the reactions in the carbon fixation pathway as a whole, instead of pyruvate specifically.

The remaining significantly enriched pathway was unexpectedly the methane pathway. Upon further investigation, it was discovered that many genes expressed in the methane pathway were also expressed in other pathways. For example, both glycolaldehyde dehydrogenase (ALDA) and 6-phosphofructokinase 1 (pfkA) are in the pentose phosphate pathway while (2R)-3-sulfolactate dehydrogenase (comC) is also found in the cystein and methionine metabolism pathway where it takes part in reactions that make pyruvate. The overlap of genes between gene sets can cause problems with detection, especially if some of the genes has a particularly strong signal. In this case, the genes in the pentose phosphate pathway have strongly defined differences in gene expression that may be masking the difference in gene expression of other genes. Although it is fairly reasonable for some genes to be present in multiple pathways, it should be checked if the overlapping genes are making biased contributions. The effect is further amplified in our data as the number of annotated genes are few.

### Enriched Pathway Plots

**Figure 1:**
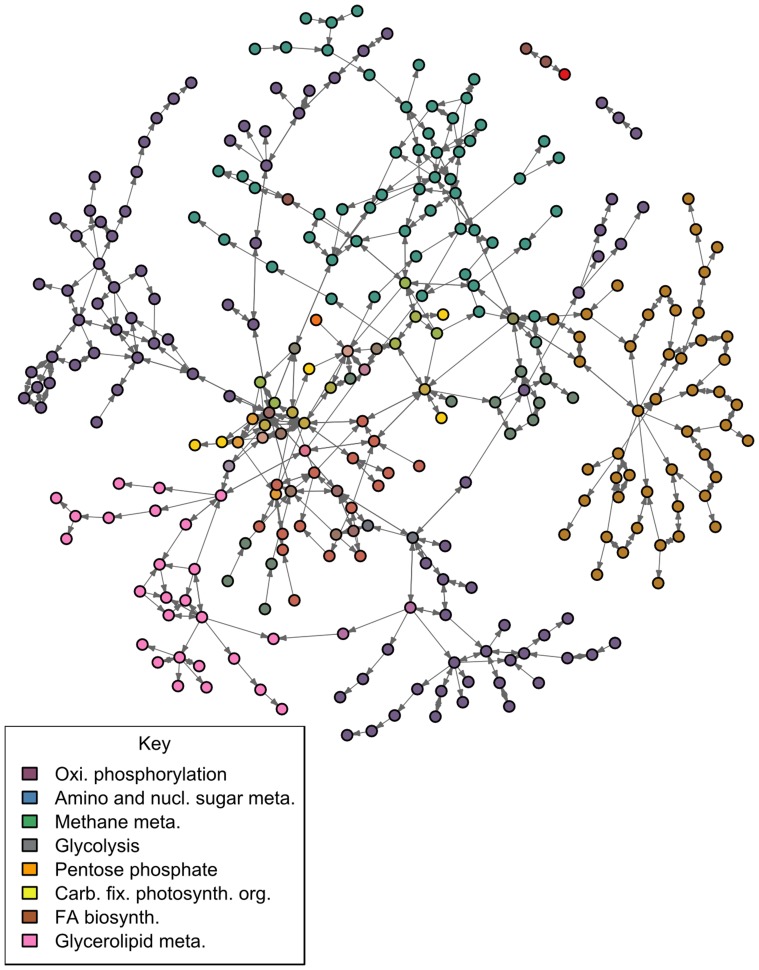
The graph of the significantly enriched pathways found using our GSEA method combined with the glycerolipid pathway. The full network contains 307 compounds and 558 reactions but compounds without reaction data were not drawn to reduce clutter. The graph is plotted with compounds drawn as nodes and reactions drawn as edges. The compounds are colored by their pathway membership; compounds belonging to 2 or more pathways are a mixture of the pathway colors. There were 7 compounds belonging to three pathways, 15 compounds belonging to two pathways and 117 compounds that were unique to their pathway. Many of the shared compounds are concentrated in the center of the graph and are related to glycolysis and pentose phosphate metabolism.

To better visualize the results from GSEA, we plotted the enriched pathways as graphs (Figure 1). The graph's nodes were set up as compounds as we wanted to focus on compounds and reactions instead of the usual approach using genes. As such, the glycerolipid pathway was added so that the key compound, TAG, was included. The graph consisted of 353 compounds and 661 reactions. Most compounds were unique to their pathway but there were 18 compounds that were found in two pathways and 13 compounds that were found in three pathways. These included pyruvate, oxaloacetate and ADP and were found in glycolysis, pentose phosphate metabolism and other related processes.

**Figure 2:**
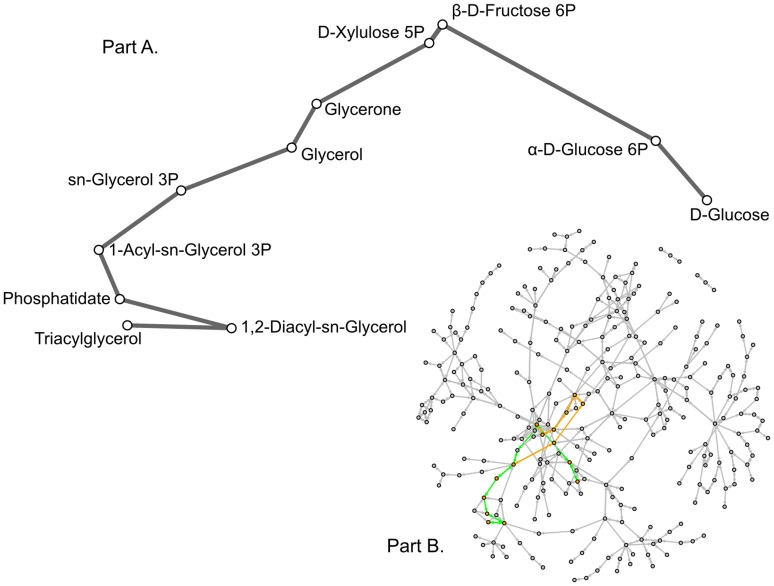
The first shortest path found in our graph between glucose and triacylglycerol using breadth-first search. A. This is the detailed view of the path showing the names of the compounds involved at each step. B. The shortest path is highlighted in green on the full graph to show its location. In contrast, the path presented in KEGG is highlighted in orange. The shortest path contains 11 compounds while the KEGG path contains 15 compounds.

**Figure 3:**
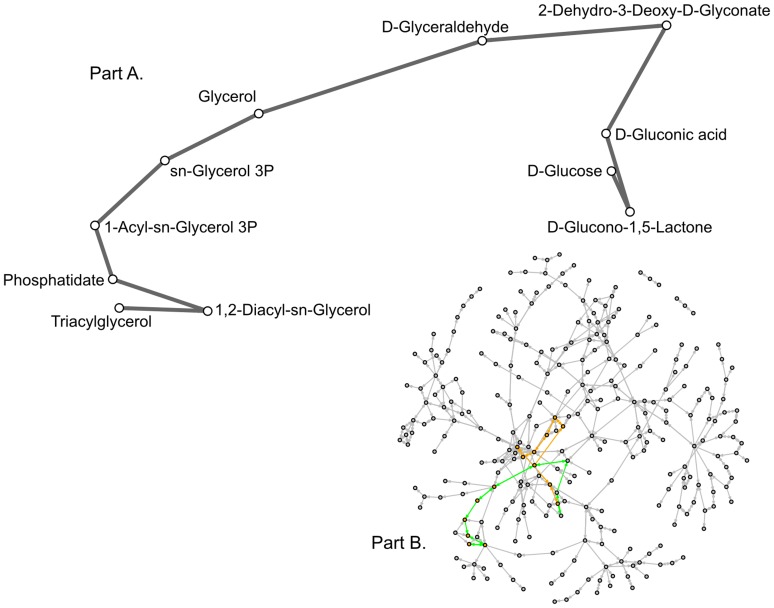
The second shortest path found in our graph between glucose and triacylglycerol using breadth-first search. A. This is the detailed view of the path showing the names of the compounds involved at each step. B. The shortest path is highlighted in green on the full graph to show its location. In contrast, the path presented in KEGG is highlighted in orange. The shortest path contains 11 compounds while the KEGG path contains 15 compounds.

Once the graph was constructed, the shortest path between glucose and TAG was calculated. As the graph was created using pathways that showed a significant relationship with oil accumulation, it can be considered a hypothesized path of metabolic reactions that metabolises glucose to produce TAG. We found two shortest paths with a length of 11 compounds (Figures 2 and 3); the conventional path found in KEGG contains 15 compounds. Our two shortest paths were very similar to each other, mainly differing between the use of glycerol or glycerone. Although it is possible to produce TAG in a smaller number of steps, it is unknown where the reactions take place in the cell. If the proteins are located close to each other, the path that was identified could be how *Fistulifera* sp. strain JPCC DA0580 produces TAG from glucose. Future experiments on metabolite quantity could also provide adequate evidence for the hypothesis.

**Figure 4:**
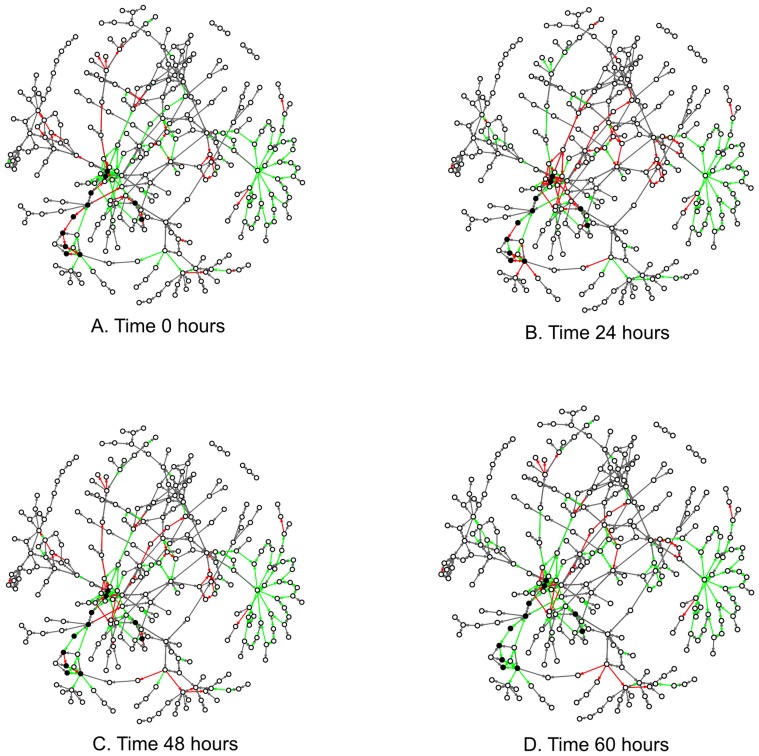
These graphs highlight the fold change direction of known genes in our data in response to oil accumulating conditions at each time point. A gene involved in a reaction is represented by an edge while the compounds in a reaction are represented by the nodes. Genes that were up-regulated during oil accumulation are drawn as green edges while red edges represent genes that were down-regulated. Genes for which data was unknown were drawn as gray edges. The compounds colored in black are part of the first shortest path found between glucose and triacylglycerol (Figure 2). The edges that connect those compounds shift from red to green during the 60 hour course of the experiment.

In the final step, we showed that the genes along the hypothesized paths were up-regulated by plotting the direction of the difference in gene expression on the edges of the graph. When viewed next to each other, the direction of the difference in gene expression at each time point shows which reactions change from up-regulation to down-regulation and vice versa (Figure 4). We observed that genes along the identified shortest paths were up-regulated during the 60 hours of the experiment. However, the up-regulation occurs in sections along the path instead of being concerted. This suggests that the gene expression of a phenotype does not change for every gene along the reaction path at a single time point. Instead, the change in gene expression occurs in sections which eventually leads to the up-regulation of the full path. This visual presentation also brings to attention the possibility of time lag effects where there could be little difference in expression in earlier time points and not others. As our method does not address this issue directly, the testing may be underpowered at detecting true signals. The testing could be improved by applying a restriction on the difference in fold change between time points or restricting time points to those where fold change differences exist. However, this would require more knowledge about the organism than we currently have available.

## Conclusion

GSEA is a useful tool for exploring data when there is a preconceived area of interest such as oil accumulation for our data. The way it can be used to analyse data more broadly is a big advantage when the data set is limited. As the cost of high-throughput sequencing experiments is decreasing, investigations with new organisms and time-course experiments can be utilized more often. For our expression data, we wanted to include time as a variable in our analysis so we modified GSEA to use it instead of removing it by averaging them. Although the number of replicates in our data caused issues with accurately isolating experimental and biological effects, we were still able to extract meaningful information through our use of resampling and GSEA. Being able to keep the time variable is an important step for future investigations. Drawbacks observed during our analysis included overlapping elements between gene sets, the reliance on pre-existing knowledge of our organism and as a consequence, the inability to assign meaning to unannotated data and improve our method's accuracy.

The results from GSEA were then graphed to produce a clear visualization of the results that is easier to interpret and grants access to other approaches for understanding the data. By plotting the direction of the difference in gene expression on our graph, we were able to observe the change in direction of the difference in gene expression as they occurred during the experiment. Using graphs in this way makes existing graph tools available, extending the investigation beyond the initial GSEA. In this analysis we looked at the shortest path of reactions between two compounds but betweenness indexes can also be investigated to identify bottleneck compounds that are important in the network. These methods can be used to help generate hypotheses as a basis for further investigations.

## Methods

### Data preparation

The expression data was gathered from *Fistulifera* sp. strain JPCC DA0580 grown in two substrates; the treatment substrate was artificial sea water where oil accumulation took place, and the control substrate was a 10 fold dilution of the treatment substrate where oil was not accumulating [19]. The RNA-Seq data was obtained at four time points (0, 24, 48 and 60 hours) when *Fistulifera* sp. strain JPCC DA0580 was grown in the two substrates. Sequences with RPKM values of 0 for all time points were discarded leaving a remainder of 22,550 sequences. We used Ssearch with MIQS [20] to annotate the sequences so that 7,822 sequences were annotated with a KEGG Orthology identifier (K ID). The unannotated sequences either did not have a match in the KEGG database or the match did not have a KEGG Orthology identifier. The gene expression of the annotated sequences were then averaged if their matching K ID was shared among several sequences, by using the following equation
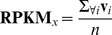
(1)where 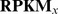 is a vector of RPKM values at each time point for K ID 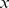, 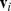 is the 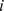 th vector of RPKM values for K ID 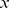 and 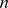 is the number of RPKM vectors with K ID 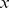. For our data, this resulted in 2,873 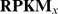
's where each vector had a length of four that corresponded to the four time points, 0, 24, 48 and 60 hours.

As RNA-Seq data often have a disproportionate amount of small RPKM values, they are usually not normally distributed, even with the use of log transformation. The resulting fold changes calculated from them can follow the same non-normality. We corrected the RPKM values by implementing a threshold of 0.1 to minimize the influence of small read numbers [21]. This was done using the sRAP R package which also performed a log transform during the normalization process [22]. The normalized RPKM vectors, 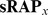, were then used to calculate the log fold change for each K ID 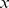 by the following equation
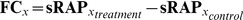
(2)where 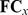 is the log fold change vector of K ID 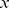, 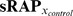 is the vector of control RPKM values of K ID 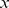 and 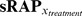 is the vector of treatment RPKM values of K ID 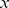.

### Gene Set Enrichment Analysis

We first established the gene sets which would be used in the analysis. Generally, gene sets are lists of gene identifiers that share an attribute of interest. For our analysis, these were K IDs divided into each metabolic pathway in the KEGG database. The pathways we chose to investigate were associated with carbohydrate (15 pathways), energy (8 pathways) and lipid metabolism (17 pathways). The Secondary Bile Acid Biosynthesis gene set was removed as our data contained no data for it, thus our analysis used a total of 39 gene sets [23] [24]. Importantly, these 39 gene sets included the glycolysis and glycerolipid metabolic pathways which contains the compounds central to oil accumulation, glucose and TAG.

The following steps of the algorithm were carried out for each gene set which produces a test statistic and p-value that describes the significance of the gene expression of the gene set compared to the overal gene expression.

**Step 1: Create a matrix of fold change data of genes present in gene set** 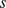 **.**

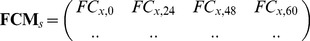
(3)where 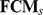 is a 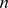 x 4 matrix, 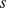 denotes gene set 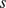, 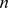 is the number of genes in the set and 4 is the number of time points in our data. Each row of 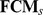 corresponds to a fold change vector 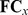 (Equation 2). This vector consists of 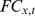 which is the fold change of K ID 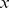 at time 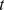. In our data, 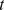 takes a value from time point 0, 24, 48 or 60 (hours).

**Step 2: Calculate the column mean of** 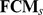 **.**

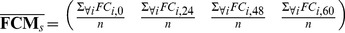
(4)where 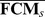 is a column mean vector of matrix 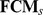 (Equation 3). This is used to represent the fold change of gene set 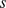 through the 4 time points.

**Step 3: Resample** 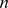 **rows from the whole fold change data matrix to construct a new matrix**, 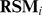. The resulting matrix, 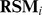, is the 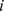
th matrix created from randomly resampling fold change vectors without replacement [25]. It has the same dimensions as 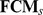 (Equation 3) but the rows of 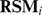 do not necessarily overlap with rows in 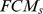.

**Step 4: Calculate the column mean of** 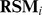 **.** The column mean 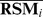 is used to represent the background fold change of 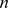 genes and is calculated in a similar manner as equation 4.

**Step 5: Repeat steps 3 and 4 6000 times.** The 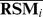 from iteration 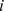 are stored as rows in a 6000 x 4 matrix, 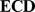.

**Step 6: Calculate the enrichment p-value of gene set** 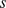 **by using an empirical cumulative distribution derived from the 6000 x 4 matrix** 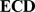 **.** The empirical cumulative distribution is defined by the following function
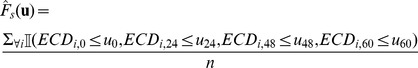
(5)where 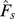 is the empirical cumulative distribution of gene set 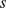, 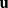 is a fold change vector with a length equal to the number of columns of 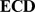 (Step 5), 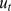 is a value in 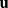 at time 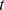 which takes the values 0, 24, 48 and 60 in our data, 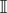 is the indicator matrix, 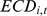 is the fold change value of the 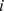
th row at time 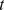 in the 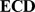 matrix and 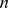 is the size of gene set 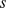.

The enrichment p-value of gene set 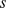 is calculated by substituting 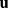 with 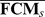 (Equation 4).

The algorithm detailed above was implemented in R [22], and the empirical cumulative distribution and enrichment p-value was calculated using the mecdf package [26].

### Enriched Pathway Plots

The significantly enriched gene sets selected from the GSEA results are metabolic pathways which were plotted to display the GSEA results and visualise reactions of the compounds within them. The generic pathway and enzyme KGML files were downloaded from KEGG and read into R. They were parsed using the KEGGgraph package [27] using the default data structure where nodes represent KEGG orthologs and edges represent reactions. This was restructured so that the nodes represent compounds and the edges represent KEGG orthologs. The graphs were then merged into one and converted into an igraph object for plotting and access to network analyses such as *get.all.shortest.paths* [28]. Unconnected nodes were removed to reduce clutter in the final plot.
